# High continuity of forager ancestry in the Neolithic of the eastern Maghreb

**DOI:** 10.1038/s41586-025-08699-4

**Published:** 2025-03-12

**Authors:** Mark Lipson, Harald Ringbauer, Giulio Lucarini, Nabiha Aouadi, Louiza Aoudia, Lotfi Belhouchet, Olivia Cheronet, Ariane-Rym Dahmani, Francesco Genchi, Francesco La Pastina, Michaela Lucci, Henry de Lumley, Nabila Mansouri, Alessia Nava, Fatma Touj, Swapan Mallick, Nadin Rohland, Alfredo Coppa, Ron Pinhasi, David Reich

**Affiliations:** 1 Department of Human Evolutionary Biology, Harvard University, Cambridge, MA, USA.; 2 Department of Human Evolutionary Biology, Harvard University, Cambridge, MA, USA.; 3 Department of Archaeogenetics, Max Planck Institute for Evolutionary Anthropology, Leipzig, Germany.; 4 National Research Council of Italy, Institute of Heritage Science (CNR-ISPC), Rome, Italy.; 5 ISMEO - The International Association for Mediterranean and Oriental Studies, Rome, Italy.; 6 Institut National du Patrimoine (INP), Tunis, Tunisia.; 7 UMR 7206 Éco-Anthropologie, équipe ABBA, CNRS-Muséum National d’Histoire Naturelle, Musée de l’Homme, Paris, France.; 8 Centre National de Recherche Préhistorique, Anthropologique et Historique (CNRPAH), Algiers, Algeria.; 9 Sousse Archaeological Museum, Sousse, Tunisia.; 10 Department of Evolutionary Anthropology, University of Vienna, Vienna, Austria.; 11 Human Evolution and Archaeological Sciences Forschungsverbund, University of Vienna, Vienna, Austria.; 12 Italian Institute of Oriental Studies, Sapienza University of Rome, Rome, Italy.; 13 Department of Environmental Biology, Sapienza University of Rome, Rome, Italy.; 14 Department of Biological, Chemical and Pharmaceutical Sciences and Technologies, STEBICEF, University of Palermo, Palermo, Italy.; 15 Institut de Paléontologie Humaine (IPH), Fondation Albert-1Er Prince de Monaco, Paris, France.; 16 Centre Européen de Recherches Préhistoriques de Tautavel (CERPT), Tautavel, France.; 17 Department of Odontostomatological and Maxillo Facial Sciences, Sapienza University of Rome, Rome, Italy.; 18 Department of Genetics, Harvard Medical School, Boston, MA, USA.; 19 Broad Institute of Harvard and MIT, Cambridge, MA, USA.; 20 Howard Hughes Medical Institute, Harvard Medical School, Boston, MA, USA.; 21 Department of Evolutionary Anthropology, University of Vienna, Vienna, Austria.; 22 Department of Environmental Biology, Sapienza University of Rome, Rome, Italy.; 23 Department of Law and Digital Society, Unitelma Sapienza, Rome, Italy.; 24 Department of Evolutionary Anthropology, University of Vienna, Vienna, Austria.; 25 Human Evolution and Archaeological Sciences Forschungsverbund, University of Vienna, Vienna, Austria.; 26 Department of Human Evolutionary Biology, Harvard University, Cambridge, MA, USA.; 27 Department of Genetics, Harvard Medical School, Boston, MA, USA.; 28 Broad Institute of Harvard and MIT, Cambridge, MA, USA.; 29 Howard Hughes Medical Institute, Harvard Medical School, Boston, MA, USA.

## Abstract

Ancient DNA from the Mediterranean has revealed long-range connections and population transformations associated with the spread of food producing economies. But in contrast to abundant data from Europe, data from this key transition in northern Africa have only been available from the far western Maghreb (Morocco). We present whole genome data for nine individuals from the Later Stone Age (LSA) through the Neolithic in Algeria and Tunisia. The earliest cluster with pre-Neolithic people of the western Maghreb (~15000–7600 BP), showing that this “Maghrebi” ancestry profile had a substantial geographic and temporal extent. At least one individual from Djebba (Tunisia), dating to ~8000 BP, harbored ancestry from European hunter-gatherers, likely reflecting early Holocene movement across the Strait of Sicily. Later Neolithic people from the eastern Maghreb retained largely local forager ancestry as well as smaller contributions from European farmers (by ~7000 BP) and Levantine groups (by ~6800 BP), and were thus far less impacted by external gene flow than other parts of the Neolithic Mediterranean.

A key event in the formation of human populations of North Africa [1–3] was the Neolithic transition to food producing economies [4], but the movements of people associated with these changes have been unclear in the absence of ancient DNA. On the European side of the Mediterranean, first farmers with roots in Anatolia expanded rapidly along the coast as far as Iberia ~7500 years before present (BP), absorbing 0–30% Western European Hunter Gatherer (WHG) ancestry along the way [5], [6–8]. These people produced an impressed style of pottery (“Cardial” in southern France and Iberia), which is also found in western Maghreb (Morocco), the only part of Neolithic North Africa for which genome-wide data has been reported[7]. However, Cardial pottery is not known from the eastern Maghreb (Tunisia and northeastern Algeria), underscoring how patterns in the west cannot necessarily be generalized to the east.

The first whole genome data from North Africa came from the ~7000 BP site of Ifri n’Amr o’Moussa in the western Maghreb (IAM) [10], where individuals derived nearly all their ancestry from a “Maghrebi” gene pool, derived from an Epipaleolithic population that also included much earlier (15000–14000 BP) individuals from the site of Taforalt (TAF; Iberomaurusian culture) [11]. However, European farmer migrants did have a large impact on closely neighboring and nearly contemporaneous populations, contributing ~80% of individuals from the ~7200 BP site of Kaf Taht el-Ghar (KTG) [12]. Within another millennium, a new component, related to Neolithic populations from the Levant, and hypothesized to have been derived from an expansion of early pastoralist societies from southwestern Asia, also appeared, constituting as much as ~50% of the ancestry of individuals from the site of Skhirat-Rouazi (SKH; ~6400 BP) [12]. All three components (Maghrebi, European farmer-related, and Levantine) contributed to individuals from the Late Neolithic site of Kehf el Baroud (KEB; ~5700 BP) [10, 12].

In the eastern Maghreb, archaeological evidence documents a distinct early Holocene (pre-Neolithic) cultural tradition, the Capsian [13, 97]. Capsian communities occupied large open-air shell-midden sites, with fewer rock shelters, and practiced hunting and gathering, focusing on terrestrial mollusks, large herbivores, and wild plants. Cultural connections extended west, east, and even north [4, 13–18]. The early Neolithisation process incorporated domesticated animals of probable Levantine origin, but otherwise retained many elements of the previous Capsian economy [15, 17]. At Doukanet el Khoutifa (DEK; Tunisia), for example, bones from domesticated caprines and (in lower number) cattle are present (especially after ~7000 BP), but domesticated plants found east and west in northern Africa are absent, and several elements reminiscent of the Capsian period remain (lithic technologies, ornaments, tooth avulsion, and consumption of terrestrial mollusks) [4, 15, 17, 19]. Pottery styles are mixed, with some but not all similar to impressed designs found in other parts of the Mediterranean [4].

It is unknown whether the eastern and western Maghreb followed similar genetic trajectories during the Neolithic. Some have argued that farmers could have crossed the Mediterranean from Sicily to Tunisia and expanded westward from there [20], although domestic plant and animal species were introduced to the western Maghreb from Iberia and the genetic data support a primary contribution from Iberian farmers [12, 17, 21]. The importance of genetic data from the eastern Maghreb is further emphasized by analysis of Mean Measure of Divergence (MMD) of dental morphological data: while Neolithic Maghreb populations are most similar to contemporaneous groups from the northern Mediterranean coast, they also show morphological affinities to Iberomaurusian populations or Natufian populations from the Levant [22].

We generated high-quality genome-wide data (most directly radiocarbon dated) for eight individuals from three sites in present-day Tunisia, and one from Afalou Bou Rhummel (ABR) in Algeria (~15000–11000 BP), an Iberomaurusian site that earlier analysis showed shared maternal ancestry with TAF [23] (Fig 1; Table 1 [TO BECOME EXTENDED DATA]; Methods; Table S1). Both individuals from Djebba (Tunisia) have earlier dates (~8000 BP) than expected for a site assigned to the Neolithic [24, 25]; we refer to them here as late Capsian, as they are near the temporal boundary of the Capsian and the Neolithic (Methods). From DEK, we obtained dates for four individuals, ranging from ~7000 to ~6350 BP. The one individual from Hergla (Tunisia) yielded a surprisingly recent date (~5900 BP) from a site only known to be occupied during the Capsian (see Methods) [26], showing that the site continued to be used by mid-Holocene groups (at least to bury their dead), a practice attested in other Capsian sites [17, 27].

Authenticity metrics indicated minimal contamination (Table S1), except for individual I13901 (ABR), for whom we restricted analyses to molecules with evidence of damage characteristic of ancient DNA (Methods). Sequencing coverage ranged from ~0.4–6.4x, measured at ~1.15 million SNPs on chromosomes 1–22 targeted for in-solution enrichment (0.05x for I13901 after damage-restriction). We analyzed these individuals together with published data to study the population history of the central portion of northern Africa before and during the Neolithic period and to trace connections to broader trends in the Mediterranean region at this time.

## Results

**Figure 2:**
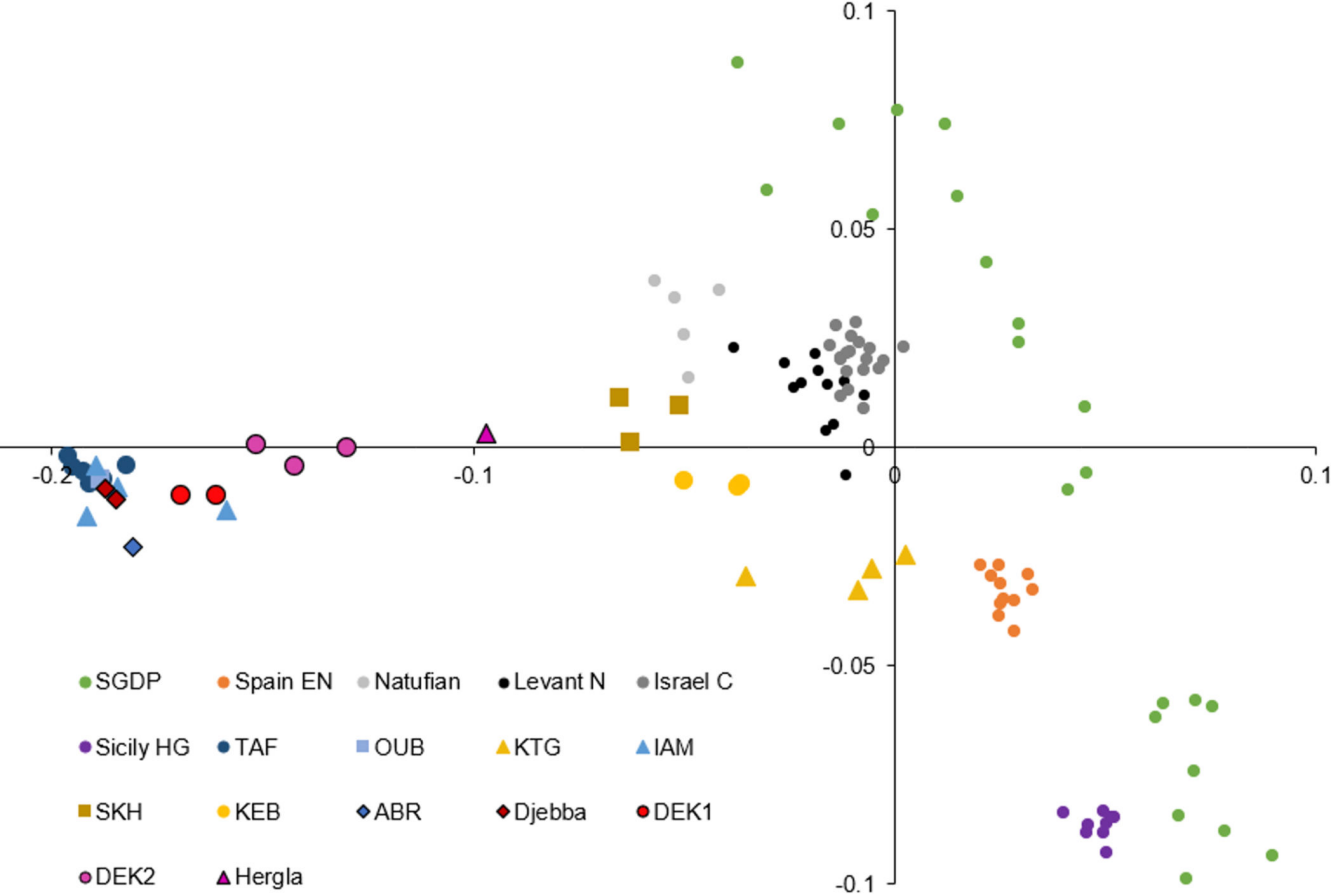
PCA results (PC1 on the x-axis, PC2 on the y-axis). The symbols for northern African ancient individuals follow Fig 1 (new data from this study with black outlines). N, Neolithic; EN, Early Neolithic; C, Chalcolithic; HG, hunter-gatherers (other abbreviations are defined in the main text). Some present-day (SGDP) individuals fall outside the displayed axis range and are omitted for readability.

We carried out a principal component analysis (PCA) as in [12], using 33 present-day individuals from 16 populations [28] from the greater Mediterranean region to compute the axes and projecting ancient individuals (Fig 2). The newly reported ancient individuals fall in the same triangular region defined by previously published ancient individuals from the western Maghreb. The three earliest eastern Maghreb individuals—from ABR (I13901, earlier than 10000 BP) and Djebba (I20824 and I20825, around 8000 BP)—cluster with people of the pre-Neolithic western Maghreb (TAF and Ifri Ouberrid [OUB]). The later individuals from the eastern Maghrem all show greater affinity to the ancient Levantine people, and Neolithic people from the western Maghreb sites of SKH and KEB.

We tested models of ancestry using the qpAdm software [6].

**Figure 3:**
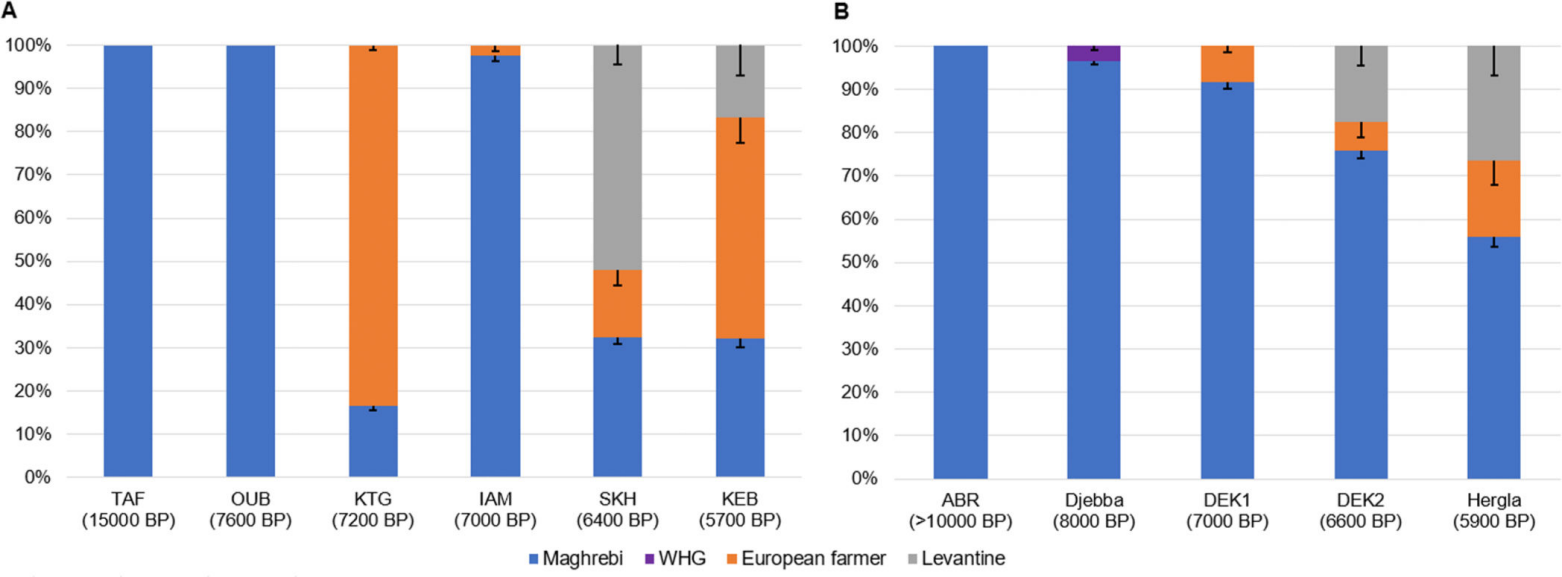
qpAdm results for (A) previously published ancient groups from the western Maghreb, and (B) newly reported groups from the eastern Maghreb, with approximate average dates for each site. Bars show one standard error for each ancestry component. For DEK2 and Hergla, the models were fit with DEK1 or DEK2 (respectively) as one proxy source, but we show standard errors obtained from a direct three-way model with Maghrebi, European farmer, and Levantine ancestry. Sample sizes (from left to right) are (A) n = 1, 3, 4, 2, 3, and (B) n = 1, 2, 2, 3, 1.

We began by reanalyzing published data from the western Maghreb (Fig 3A; Table S2), using individuals with no apparent recent admixture (either TAF or later individuals) as a proxy for Maghrebi ancestry; Early Neolithic individuals from Spain as a proxy for European farmer ancestry; Chalcolithic-period individuals from Israel as a proxy for Levantine ancestry; and a set of outgroups chosen to distinguish among these three components (Methods). The results are similar to those previously reported [10, 12], with the exception that we find evidence for a small amount of European farmer ancestry (although our power is not sufficient to rule out a different source) among the Early Neolithic individuals from IAM, previously described as having 100% Maghrebi ancestry. We infer 9.6±2.5% European farmer ancestry for IAM.7 (the farthest right of the four IAM individuals in the PCA, Fig 2), and 4.7±2.0 % for iam004; these two individuals are slightly more recent (by ~200 years) than the others published from the site. Additionally, whereas the Middle Neolithic individuals from SKH were previously modeled with a two-way mixture of Maghrebi and Levantine ancestry, we find that such a model is insufficient (p-value < 0.05; see Methods), with signals of excess ancestry related to European farmers or to European hunter-gatherers (using reference individuals from Spain and Serbia, who derive most of their ancestry from the “western hunter-gatherer”, or WHG, gene pool). In fact, even a three-way model with Maghrebi, European farmer, and Levantine ancestry fails to fit (p = 0.006). The results in Fig 3A are based on a model in which we use Middle Neolithic farmers from Sardinia – who have more WHG ancestry than the Early Neolithic individuals from Spain (~20% versus ~10%) – as the proxy for European farmer ancestry (p = 0.15). Although we report a three-way model for KEB as well (the same model used in previous work), the evidence for Levantine ancestry is weak (p = 0.044 for a model with only Maghrebi and European farmer ancestry).

For the eastern Maghreb, we tested similar two- and three-way admixture models (Fig 3B; Table S2). As a proxy for Maghrebi ancestry, we used the OUB individual together with one from IAM (IAM.4.5). Individual I13901 (ABR, Algeria) fits with 100% Maghrebi ancestry (p > 0.18). We also repeated the analysis with the full (contaminated) data for I13901 and obtained a nearly successful fit (p = 0.023) with Maghrebi ancestry plus a component capturing contamination from a present-day European source (1000 Genomes CEU as a proxy, proportion ~28%).

The two early individuals from Djebba also cluster with TAF and related groups in PCA. For both Djebba and ABR, we observe (via f4-statistics) distinctive allele-sharing with Natufians as previously reported for TAF [11], confirming a high degree of long-term genetic continuity leading up to the Neolithic (Table S3). But while one of the Djebba individuals (I20824, ~8100 BP; see Methods) can be fit with 100% Maghrebi ancestry in qpAdm (p = 0.1), the second (I20825, ~7900 BP) has excess affinity with WHG; direct f4-statistic allele-sharing tests are significantly nonzero at up to Z = 5 (Table S3). This cannot be explained by adding European farmer ancestry (which includes a WHG-related component) in qpAdm (p = 0.0002 or 0.0001 using Spain EN or Sardinia N as a proxy). However, we can fit a model for I20825 with Maghrebi plus 5.7±1.1% WHG-related ancestry (p = 0.36). For the two individuals as a pair (Fig 3B), the proportion is lower (3.1±0.8%, p = 0.8), and the evidence against models without WHG-related ancestry is weaker (although still marginally significant; p = 0.02–0.04).

We searched for allele-sharing signals that might indicate the possible geographic source of the European hunter-gatherer-related ancestry at Djebba (Methods). Specifically, we computed statistics to detect differential relatedness to hunter-gatherers from Sicily (Epigravettian and Mesolithic [8, 29]); Germany and the Netherlands [29]; Spain [29–33]; Serbia [34]; and Russia [29, 31]. For individual I20825, we observe (marginally) significant signals of differentiation when comparing the Sicily hunter-gatherers to those from Spain and Russia (Z = 2.1 and 2.3), lower differentiation to Serbia (Z = 1.6), and none between Sicily and northern WHG (Z = −0.1; Table S3). Given what is known about genetic structure in Mesolithic Europe [29] – including similar WHG ancestry across a wide geographic area – these observations are as expected for a very small proportion of WHG ancestry in I20825. We also used subsets of the European hunter-gatherer data to test for the possible presence of Maghrebi ancestry in Sicily but did not observe any significant signals (max Z < 1.7 out of 24 statistics computed; Table S3).

Outside of Djebba, the highest proportion of Maghrebi ancestry in any individual was ~92% in I22580 (~7000 BP from DEK), who could be fit with a mixture of Maghrebi and European farmer ancestry (p = 0.18), but not Maghrebi and Levantine (p = 0.0046; Fig 3B; Table S2). The individual closest to I22580 in the PCA is I22862 (undated from DEK; I22580 and I22862 are labeled “DEK1” in Fig 2). I22862 has a slightly higher affinity to WHG; several models with small variations in ancestry sources were successful at the p = 0.05 threshold (Table S2). When we pooled I22580 and I22862 into a single DEK1 population, a two-way model with Maghrebi and European farmer ancestry was successful (~92% and 8%; p = 0.16).

The other four Neolithic eastern Maghreb individuals – three from DEK (“DEK2” in Fig 2) and one from Hergla – fall farther to the right in PCA. I22866 (DEK) can be fit with Maghrebi plus either Levantine (p = 0.76) or European farmer (p = 0.07) ancestry (or both), while I22867 (DEK) can be fit with all three sources (p = 0.6) and nearly with only Maghrebi and European farmer ancestry (p = 0.04). I22577 (DEK) has its best fit with all three sources, but it remains sub-threshold (p = 0.01), and likewise for I22852 (Hergla; p = 0.0004). If we pool together all three DEK2 individuals, we obtain successful fits with Maghrebi plus Levantine ancestry (~76% and 24%; p = 0.14), with all three sources (including ~9% European farmer ancestry; p = 0.26), or in a two-way model using the DEK1 subgroup as one source plus Levantine ancestry (~83% and 17%; p = 0.23), which is the model shown in Fig 3B. For I22852 (Hergla), we also tested models with either the DEK1 or DEK2 subgroup as one source plus European farmer or Levantine ancestry. Several combinations had better-fit quality than the three-way model above but still slightly sub-threshold (p ~ 0.01–0.02), e.g., DEK2 as one source plus additional European farmer and Levantine ancestry (~74%, 13%, and 14%; Fig 3B, Table S2).

We performed additional analyses to investigate refinements to the qpAdm models for DEK and Hergla. First, we used f4-statistics to compare allele-sharing between the Tunisia individuals and early farmers from Spain and Italy [8, 35–38]; none of the statistics detected asymmetry (max |Z| = 1.5; Table S3). Second, for DEK2, we tested alternative models using earlier ancient Levantine groups (either Natufian [39, 40] or Neolithic Levant [39–41]) as proxies in the DEK1-plus-Levantine model. Both had lower p-values than the Chalcolithic Israel proxy source (p = 0.048 and 0.032, versus p = 0.23), despite less power to reject the model (larger standard errors; Table S2). Third, we searched for signals of additional ancestry from the Saharan region using qpAdm experiments with present-day Fulani, Laka, and Bulala populations [42] as outgroups, and did not find any significant violations of the baseline models (Methods; Table S4).

Uniparental markers are consistent with our genome-wide results in indicating a majority of Maghrebi ancestry among newly reported individuals, with most admixture from other sources later in the transect. Of the five males, four can be assigned to Y chromosome haplogroup E1b1b1a1 characteristic of northern Africa, particularly in ancient individuals with Maghrebi ancestry [11, 43]. The exception is I22852 (Hergla), who carried T1a1a, associated with Levantine farmers [39]. For mtDNA, individuals from ABR and Djebba, as well as both from the DEK1 subgroup and one from DEK2, carried subclades of U6, common in ancient north Africa [11, 44, 45]. Haplogroup L3f1b+16292 (I22867, DEK2) belongs to a clade hypothesized to have originated in eastern Africa and spread to other parts of the continent [46], while R0a2 (I22852, Hergla) has a wide distribution also observed in the Neolithic Levant [39, 40]. Finally, individual I22866 (DEK2) carried mtDNA haplogroup U5b2b1, which is characteristic of pre-Neolithic Europe [47], and, thus, likely derived from European hunter-gatherers, either directly (hunter-gatherers crossing the Strait of Sicily) or via European farmers (admixed with WHG ancestry).

We inferred admixture dates using ALDER [48] and DATES [49]. As reference populations, we used TAF plus Early Neolithic individuals from Spain, Chalcolithic-period individuals from Israel, or Mesolithic hunter-gatherers from Serbia (Methods). When testing IAM from the western Maghreb, we inferred significant signals of admixture ~8–13 generations in the past (Table S5). This provides a second line of evidence proving that our discovery of European farmer ancestry in IAM.7 is not due to contamination (which would not generate a signal of admixture linkage disequilibrium). For Djebba (Tunisia), I20825 had a significant admixture signal (using the Serbia Mesolithic reference), with a date of 18.2±3.0 generations before the individual lived from ALDER and 13.9±6.5 from DATES. We obtained a weaker, but still significant, signal for I20824 with ALDER (16.3±6.4 generations, amplitude 10±5.0 ×10^−4^, as compared to 28±5.7 ×10^−4^ for I20825), raising the possibility that both individuals from Djebba had a small proportion of WHG-related ancestry. When we used the Spain farmer reference for the Djebba individuals, the signals became weaker (19.3±6.5 generations, amplitude 17±3.9 ×10^−4^ for I20825 with ALDER; not significant for I20824; not significant for either individual with DATES, although we obtained a date of 20.2±6.2 generations for I20825 using the Israel reference). This is consistent with a WHG-related rather than European farmer-related source. We obtained relatively recent dates for the five individuals from DEK (~5–25 generations); thus admixture was happening within a couple of hundred years of the time the individuals lived.

**Extended Data Figure 1:**
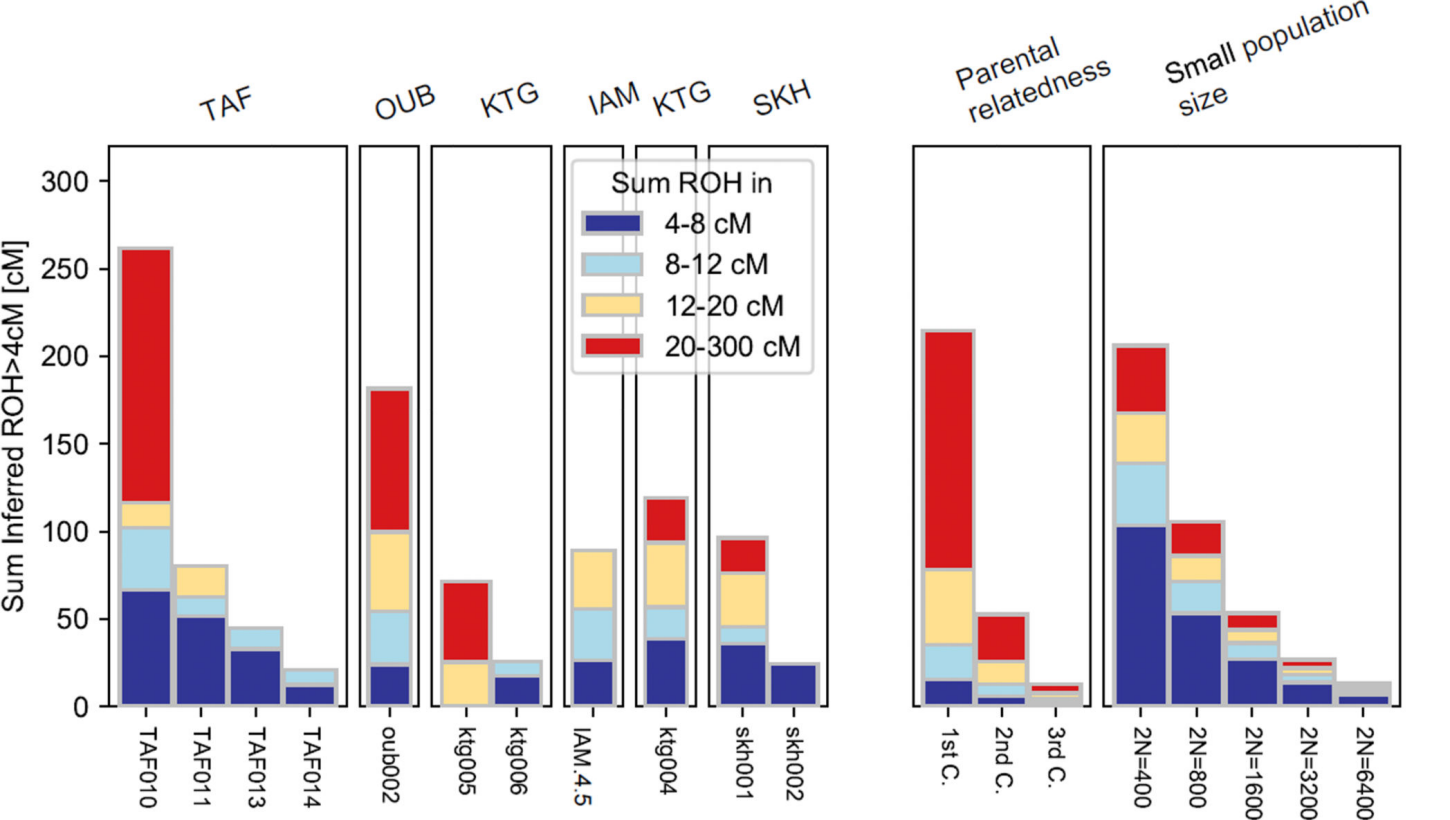
Left side, inferred runs of homozygosity (ROH) for ancient individuals from the western Maghreb; right side, expected distributions under different scenarios. Three individuals have signatures of likely parental relatedness: TAF010 and oub002 (first-cousin parents), and ktg005 (second-cousin parents).

**Figure 4:**
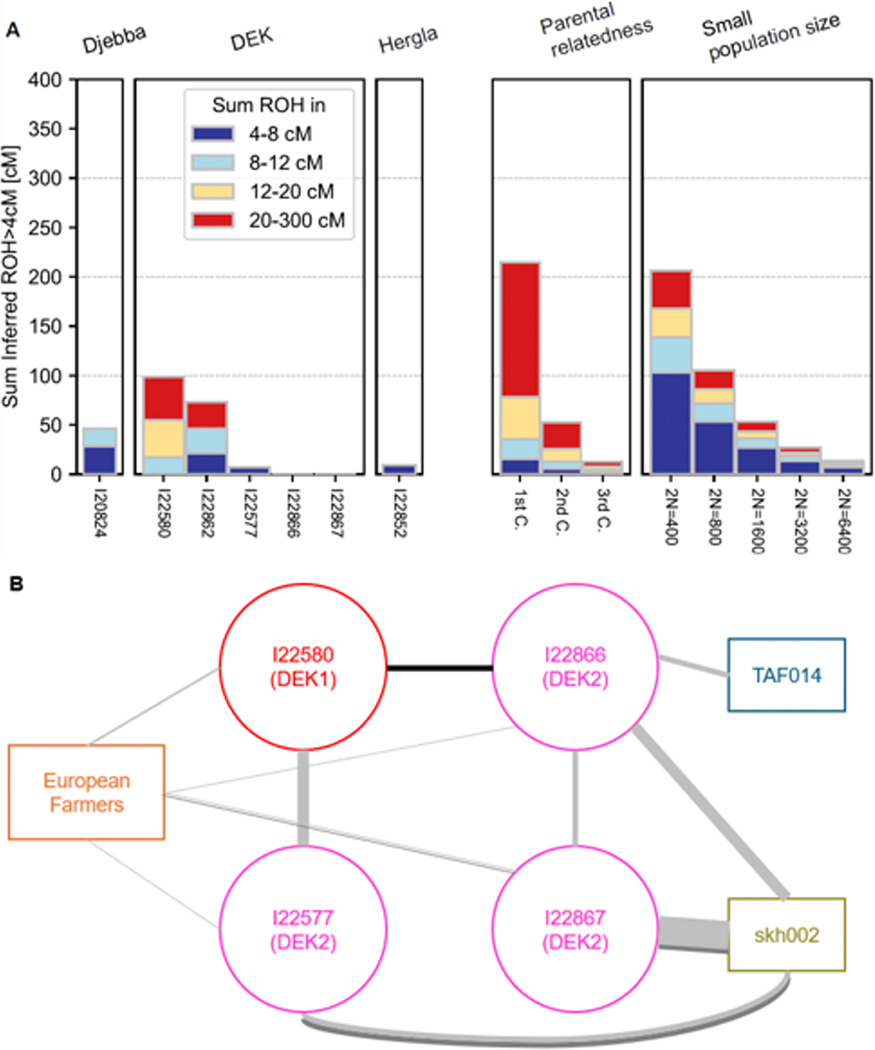
(A) Inferred runs of homozygosity (ROH). The left side shows ROH for seven eastern Maghreb individuals, while the right shows expected distributions under different scenarios. (B) Graph of inferred inter-individual IBD sharing for four of the eastern Maghreb individuals. The European farmer node represents an aggregate of 33 individuals. Darker lines indicate longer segments (black, > 20 cM; dark gray, 12–20 cM; light gray, 8–12 cM), and line thicknesses are proportional to the number of segments (normalized by a factor of 33 for sharing with European farmers).

We using *hapROH* [50] to infer runs of homozygosity (ROH) for the seven individuals for whom we had sufficient data (all except I13901, from ABR, and I20825, from Djebba; Fig 4A, Table S6). The presence of ROH can reflect both small local population sizes and close relatedness of an individual’s parents. The longest ROH and largest ROH totals are found in the two DEK1 individuals (I22580 and I22862), whose distributions suggest second-cousin parents. The next-longest segments and next-largest total belong to I20824 (Djebba), with a distribution consistent with a relatively small recent effective population size (N_e_; 95% CI 337–1401). Among the three DEK2 individuals, we inferred only one ROH longer than four centimorgans (cM), yielding an estimated N_e_ of at least ~3500. I22852 (Hergla) had small but non-zero ROH, with two segments between 4–5 cM (N_e_ 95% CI 828–15540). Overall, ROH totals in the eastern Maghreb are lower than in the west (Extended Data Fig 1) [12] where Neolithic and pre-Neolithic individuals had extensive ROH and low ancestral population sizes, similar to Mesolithic Europe [12, 29, 50].

We used *ancIBD* [51] to search for segments of chromosomes shared identical by descent (IBD) due to recent shared ancestry between the ancient eastern Maghreb individuals (four with sufficient sequencing coverage, all from DEK) and other ancient individuals (Fig 4B; Table S7). The only long (> 20 cM) segment is shared between I22580 from DEK1 and I22866 from DEK2, indicating at least some continuity at the site. We also find three medium-long (12–20 cM) segments indicating shared ancestry within ~2000 years: I22867 and I22577 from DEK2 each shares one such segment with skh002 from SKH, and I22867 shares one with a Neolithic individual from France (who also shares a shorter segment with I22866). Of 45 shorter IBD segments (8–12 cM), all involve sharing between DEK individuals and ancient northern Africans (eight between DEK and skh002, one shared with TAF, three shared within DEK) or ancient European farmers (~8000–4000 BP; Fig 4B; Table S7). We find no links to the Levant.

## Discussion

Our results broaden our understanding of the structure of pre-Neolithic populations of North Africa. The two newly sampled individuals from Djebba (Tunisia), and one from ABR (Algeria), derive from the same indigenous source as contemporaneous groups from the western Maghreb [10–12], revealing that this “Maghrebi” ancestry had a wide geographic and temporal extent. Unlike the West, however, in the eastern Maghreb, we find evidence of admixture from Western European hunter-gatherers at Djebba. The geographic proximity of Tunisia and Sicily, and evidence for seafaring and contact across the Strait of Sicily at precisely this time (~9000–8000 BP) based on material culture links (pressure technique, and obsidian from the island Pantelleria present at Capsian sites in the eastern Maghreb) [4, 17, 18], suggest the Strait of Sicily was the likely route. This cultural exchange appears to have been accompanied by the movement of hunter-gatherers, at least from north to south and possibly in both directions.

In the Neolithic, our results show that European farmer ancestry previously documented to have impacted the western Maghreb by ~7400–7300 BP [12], was ubiquitous in the eastern Maghreb after the Capsian/Neolithic boundary. The importance of the arrival of European farmers is underscored by our finding of at least a small proportion of European farmer-related ancestry at every Neolithic site, even those where it was previously inferred to be absent [10, 12].

A notable difference in ancient DNA from the western and eastern Maghreb is the presence in the west of some individuals with high proportions of European farmer-related ancestry (specifically from KTG in the Early Neolithic, with more than 80%, and KEB in the Late Neolithic, with more than 50%). In the east the maximum is less than 20%. These results are striking in light of archaeological evidence of a greater influence from European farmers in the western than in the eastern Maghreb (where no Cardial pottery has been found, and farming of domesticated crops only appears much later) [15, 17, 21]. Our results add to the weight of evidence pointing to Iberia as the primary source for the migration of farmers from Europe to the Maghreb [12, 17, 21], from which it diffused in west-to-east direction.

The other widespread admixture event involves Levantine-related populations, which in both time transects arrives many centuries later than European farmer-related ancestry. In the western Maghreb, its first appearance is at SKH (~6400 BP; oldest single individual 6730–6500 cal BP), while we find it slightly earlier in the eastern Maghreb, via I22867 and I22866 from DEK (6888–6678 and 6828–6662 cal BP). It is tempting to associate the (limited) European farmer admixture in the “DEK1” genetic cluster with the earlier stage of occupation at the site (~7400–7000 BP) and Levantine admixture in the “DEK2” genetic cluster with the later stage (~7000–6300 BP, with increased reliance on domesticated animals and more usage of typically “Neolithic” pottery and stone tools) [4, 17]. The lack of IBD segments shared between eastern Maghreb individuals and ancient individuals from the Levant, is plausibly due to a combination of both (1) limited sampling in the Levant, and (2) larger effective population sizes in this region as compared to European farmers, resulting in less extensive IBD. Domestic caprines, likely brought by people from the Levant, were first attested in the eastern portion of northern Africa by ~8200 BP and then spread westward [14, 17, 54, 55]. Although available ancient DNA sampling is insufficient to provide a fine-grained chronicle of the westward spread of Levantine ancestry, our work increases the weight of evidence that it involved passage through the eastern Maghreb, potentially mediated by movements of people between these regions that we attest directly by a medium to large IBD segment shared between DEK and SKH.

Our results show that regional demographic trajectories were highly variable in Europe and North Africa during the Neolithic transition. In Europe, virtually all populations traced most of their ancestry to early migrants from Anatolia, with smaller contributions from local hunter-gatherers, and similar admixture trajectories in each region [35]. In contrast, data from the western [10, 12] and eastern Maghreb show that northern Africa featured both (a) more heterogeneity and (b) more continuity of autochthonous ancestry after the arrival of migrants and adoption of new lifestyles and technologies. Indeed in the eastern Maghreb, the archaeological record of the Neolithic transition is more consistent with local continuity than in Europe [4, 17]. A possible explanation for the resistance of eastern Maghrebi populations to admixture from farmers is that local hunter-gatherer populations in northern Africa remained more stable and resilient than those across the Mediterranean during the ~8200 BP climatic cooling event, and also that the density of migrating farmers was lower than in Europe and the western Maghreb, perhaps because the region was less suitable for agriculture (which did not develop in the eastern Maghreb until much later) [17]. Thus, the dilution of local ancestry may have been less substantial than in Europe or the western Maghreb. Larger populations in the eastern Maghreb than in the western Maghreb—as tentatively suggested by our analysis of Runs of Homozygosity—may also help to explain why local group were more resilient to the arrival of migrants and retained relatively high proportions of local ancestry. The insights from this study highlight how much remains to be learned through ancient DNA analysis of previously unsampled places and times, and interdisciplinary study of the human past.

## Methods

### Site descriptions

#### Afalou Bou Rhummel (Algeria)

The Afalou Bou Rhummel shelter is part of a vast karstic network located in the coastal region of the Babors massif. This extends from east to west, starting from the Soummam valley to the town of Jijel. The southern slope of the massif slopes down to the high plains of the Sétif region, while the northern slope, facing the sea, forms the cliffs that today make up the Kabyle corniche. These cliffs rise to a height of between 500 and 800 m. It is on this coastal slope, along the semicircle formed by the Gulf of Bédjaïa, that the Afalou Bou Rhummel shelter opens.

The shelter overlooks the road, which runs between the massif and the sea, at a height of 40 m. It faces north-north-east and is 22 m wide and 10–12 m deep. The ceiling is convex and is pierced in the middle by a natural chimney that rises 10 m; its diameter reaches 3 m. This naturally formed chimney illuminates and ventilates the shelter.

The site was discovered by the geologist A. Ehermann in 1920, and in 1928, he began to excavate the site with Boule, Vaufrey, Reygasse and Arambourg. The survey revealed the presence of prehistoric industry, faunal remains, and numerous human remains. Arambourg conducted three excavation campaigns (1928, 1929, and 1930) [56]. This research led to the discovery of a large Iberomaurusian (LSA) occupation.

A team from the CNRPAH, directed by Hachi, resumed research at the site and carried out several excavation campaigns between 1983 and 1993. They discovered abundant archaeological material and new burials in the same levels as those at Arambourg. From the different levels, a series of dates was obtained (Extended Data Table 1) [57–60].

As the field archives from Arambourg were never found, the stratigraphic relationship between the two excavations has not been established with certainty. However, it has been speculated that the old level I is related to layer IV of the new (Hachi) stratigraphy and the old level III to the new layer X [57–60] (Extended Data Tables 1–2).

**Extended Data Figure 2:**
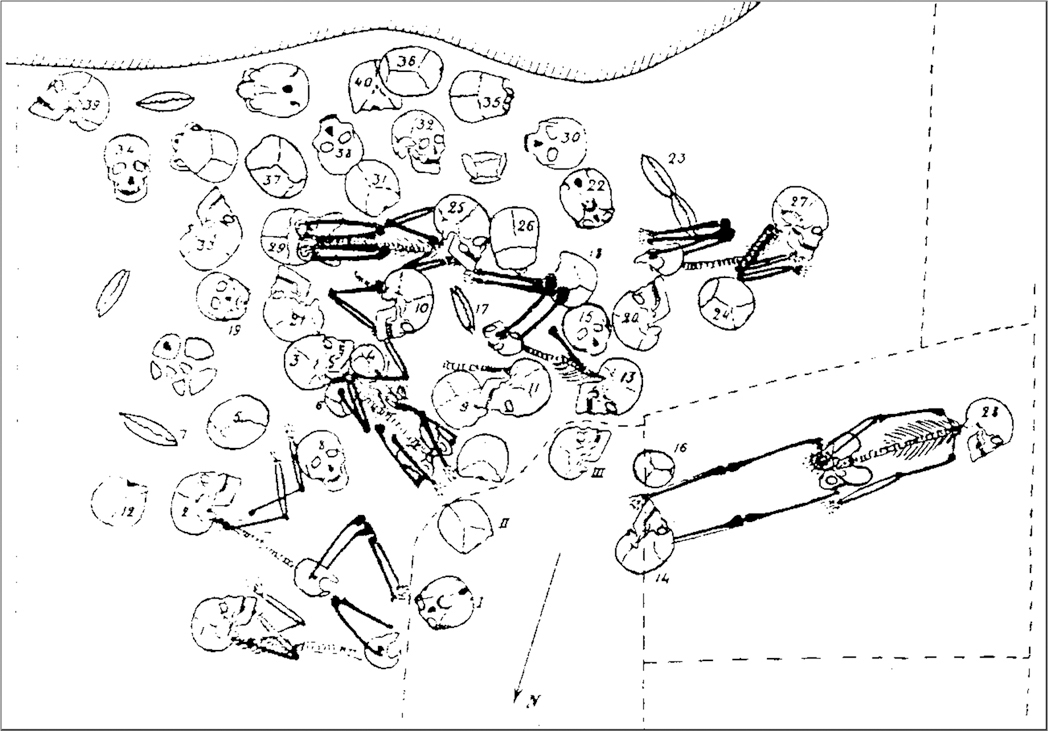
Distribution of the six connected individuals (H1-H2, H3, H13, H25, H27) and the isolated craniofacial blocks from burial A at Afalou Bou Rhummel. The individual from whom we successfully extracted DNA is H2 near the bottom left (red arrow). The recumbent skeleton, framed by the dotted line, is burial C from the lower level (H28 and H16). Modified from ref. [56].

The funerary complex at Afalou Bou Rhummel comprises two levels of burials (Extended Data Table 2; Extended Data Fig 2). In the upper level, two multiple burials were discovered. The first, which we refer to as A and which includes the individual from which we successfully extracted DNA, housed 49 individuals, of which 39 were determined to be adults, one adolescent, and 9 younger children (Arambourg excavations 1928–1932) [61] and the second, which we refer to as B, functioned as a true collective burial with an empty space and a collective grave (Hachi excavation 1983–1993). It housed eight individuals [60]. The lower level also yielded two burials. The first, labelled C (Arambourg excavations 1928–1932), housed primary individual H 28 (adult) together with individual H 16 (immature). The second, designated D/E, is a sepulchral unit containing two primary and individual deposits, H IX and H X [61, 62].

The entire Afalou Bou Rhummel collection from the Arambourg excavations is kept at the Institut de Paléontologie Humaine in Paris, except for a craniofacial block that remains at the CNRPAH in Algiers (Algeria). The material is in a very good general state of preservation, with little fragmentation. Most human remains are numbered according to the order in which they were found.

#### Djebba (Tunisia)

**Extended Data Figure 3:**
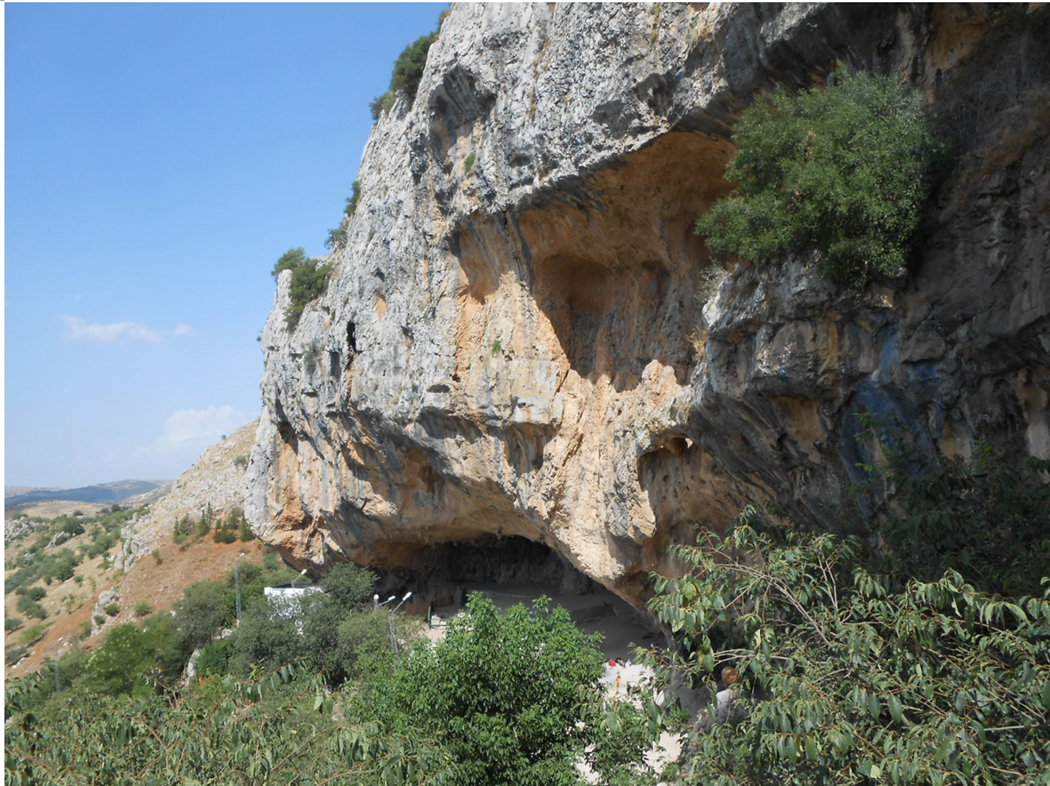
View of the Djebba Shelter.

The shelter of Djebba is located on the Goraa plateau (900 m above sea level) in the Haut Tell (Northwestern Tunisia; Extended Data Fig 3). The Goraa plateau overhangs the perched village of Djebba, which is 6 kilometers south of the city of Thibar (Beja, northwestern Tunisia), and overlooks the Medjerda plain. The huge Shelter of Djebba, cut into the Eocene limestone of Goraa Mountain, is 150 m in length and 35 m in width.

R. Vaufrey discovered the site in 1927, opening three trenches from the top to the bottom of the *rammadiya* (ashy mound) deposits. Despite the poor archeological material, Vaufrey categorized the shelter as Neolithic [22]. In 1978, J. Zoughlami (INP, Tunisia) opened a test trench in the middle of the shelter [63]. The material exhumed, mainly composed of lithic artifacts, includes microlithic elements and geometrics, among which trapezes are the most frequent [52, 64].

In 2018, new fieldwork was undertaken in the site led by co-author N. Aouadi (INP, Tunisia) and in the dolmen of the Goraa plateau to understand differences and continuity in the occupations of the Goraa Mountain [53]. In the shelter of Djebba, five test trenches SDJ1, SDJ2, SDJ3, SDJ4, and SDJ5 were opened in different areas. The site is a typical *rammadiya* deposit consisting of a mound of ashes, burnt stones, land snail shells, faunal remains, lithic artifacts, Unio shells, pottery, and ostrich beads. The faunal remains belong to wild taxa, such as gazelles and aurochs, and domestic taxa, such as caprines and cattle.

In SDJ2, human remains from two individuals were uncovered: Skeleton 2 (I20824), from the lower part of the sequence (SU6b), showed avulsion of both maxillary and mandibular central incisors, as well as one of the two mandibular lateral incisors. This individual has been dated to approximately 8200–8000 cal. BP (Table 1; Table S1). Skeleton 1 (I20825), from SU4, was dated to 8000–7800 cal. BP. Given the lack of a confirmed stratigraphic association between the two individuals and the domestic faunal remains, it is plausible that they belong to an earlier phase of occupation, potentially dating to the late Capsian period or situated near the transitional boundary between the Capsian and Neolithic.

#### Doukanet el Khoutifa (Tunisia)

Doukanet el Khoutifa is a Neolithic site located on a series of superposed terraces about 700 m above sea level along the El Garia crest of the Tunisian Ridge. The upper and main terraces occupy a platform of ~ approximately 2,200 square meters.

All the occupation layers were attributed to the Neolithic. The first series of radiocarbon dates assigned the sequence to a period ranging from the second half of the eighth to the early seventh millennium BP. New dating, done directly on the skeletons analyzed in the present research, confirms previous data and places the cemetery in this same time frame (Table 1).

**Extended Data Figure 4:**
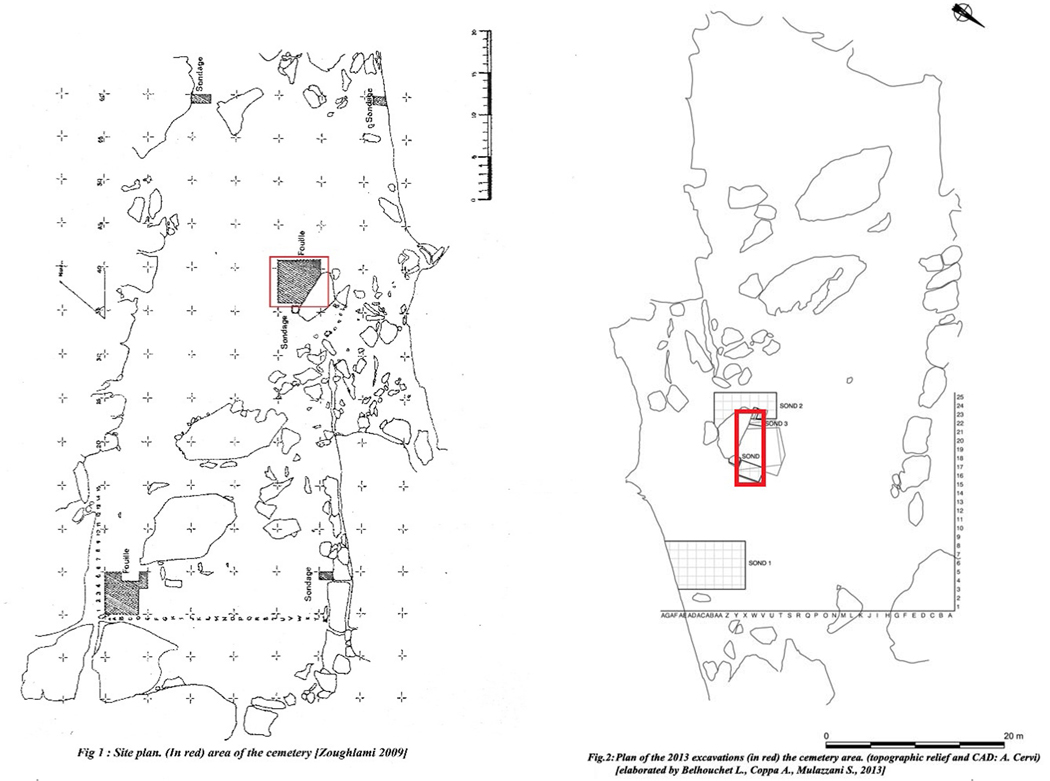
Site plans from Doukanet el Khoutifa (DEK). (A) Original 1970s excavations. (B) Excavations from 2013.

The site was mentioned for the first time by L. Balout in 1955 [65], and J. Zoughlami later explored it in 1972–1973 and 1976 [52, 63], excavated four trenches, which revealed the presence of a structured village and a cemetery organized around a massive rock at the center of the terrace (Extended Data Fig 4A).

The resumption of the archaeological investigations in 2013 [66] saw the creation of a new topographical plan (Extended Data Fig 4B), the opening of a survey in the eastern quadrant of the site of approximately 8×5 m realized to understand the structures and spatial layout of the site (*sondage 1*), and further investigations in the area identified as the cemetery (*sondages 2–3-4).*

Field activities at the site were carried out in 2013, 2018, and 2022, with the excavation of new trenches, to better clarify the chrono-cultural sequence and occupational patterns and verify the spatial organization between the living and burial areas. Analysis of faunal and floral remains confirmed the presence of domestic animals, among which were cattle and caprines, but did not reveal the presence of domesticated plants [4].

From the original excavations in 1972–1973 and 1976 [52, 63] in the area corresponding to sector 2 of the excavation, the minimum number (calculated on the basis of teeth) of individuals buried is 18, with the post-cranial bones attributed with certainty to 10 of these [52, 67]. It was possible to distinguish 2 infants, 3 juveniles, 12 adults, and 1 older adult individual.

The determination of the sex of morphological adults is more complex due to the fragmentary nature of skeletal remains. However, 60% of skeletal remains are attributed to males and 30% to females [52]. The positions of the head were generally toward the north. In some cases, a circle of stone was documented around the deposition, which could be interpreted as part of the burial [52, 67]. A further four individuals were identified and partially studied during the 2013 excavations, and then fully exhumed during the 2018 and 2022 excavations.

#### Hergla (Tunisia)

Hergla (SHM-1) is an Upper Capsian open-air site located in the Hammamet Gulf, on the Eastern Tunisian coast [26]. It occupies an Early Holocene hydro-aeolian dune formed during an arid episode, on the western edge of the Halk el Menjel sebkha-lagoon, about 3 m above the current sebkha (salt flat).

The site was discovered by E.G. Gobert in 1954 and was first excavated between 1969 and 1971 by M. Harbi-Riahi and J. Zoughlami [68]. Seven new trenches covering a total of 110 sq. m were excavated between 2002 and 2007 within the framework of a joint Italian-Tunisian scientific agreement (University of Bologna; Istituto Italiano per l’Africa e l’Oriente of Rome; Institut National du Patrimoine of Tunis).

The geomorphological and pollen analysis carried out on the sediments of the site and around the sebkha allowed reconstruction of climatic evolution and local environmental changes. The sebkha-lagoon system was fed by an impressive river supply and characterized by the presence of abundant fishing resources.

The micromorphological analysis of the sediments highlighted an uninterrupted anthropogenic sequence, confirming the continuity of the occupations and the sedentary lifestyle of the groups living in the area.

The site consists of seven occupation layers that can be divided into two main phases. The first phase, from layers 1 to 4, is dated to the 9^th^ millennium BP, and the second, from layers 5 to 7, is dated to the first half of the 8^th^ millennium BP.

Numerous remains of buildings are present in all the occupation layers: storage structures, cooking features, fireplaces, remnants of walls, and possibly post holes.

Archaeological analysis suggests that the economy was largely based on hunting, fishing, and gathering activities. No remains of domestic fauna were recognized, but a significant variability of exploited species were observed: gazelle, giraffe, rhinoceros, wild boar, small mammals, and birds. During the second phase of occupation, there is evidence of intensive exploitation of bovids, including *Bos primigenius*, *Alcelaphus buselaphus*, *Gazella dorcas*, and *Gazella cuvieri*. Domestic plants are also absent.

The site also revealed the presence of pottery starting from the early 8th millennium BP. The decorative motifs recall the impressed ceramics that developed at the same time in the central Mediterranean. In the second phase of occupation, the site also yielded a number of artifacts manufactured with obsidian from Pantelleria.

Two primary burials have been identified at the site, namely Burial 1 (lacking a skull) and Burial 2 (skull present), as well as fragments bringing the total to at least four individuals: A) a 4–6-year-old child; B) a 1–2-year-old child; C) a rather robust adult (Burial 1); and D) a rather frail adult (Burial 2) [69, 70].

**Extended Data Figure 5:**
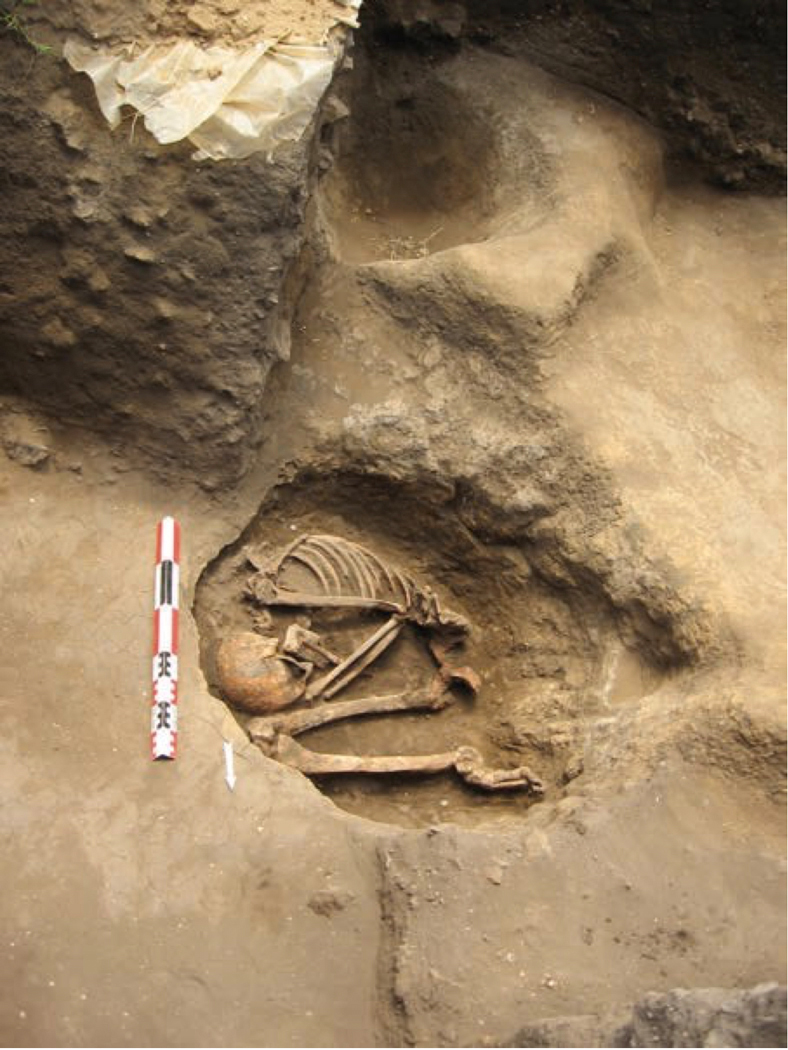
Burial 2 from Hergla (SHM-1)

The grave filling of Burial 2, the individual successfully sampled for ancient DNA for this study (Extended Data Fig 5), is dated on a mollusk (*Cerastoderma glaucum*) as 8269–7762 cal BP (7595±80 BP, Pa 2471). However, the direct dating on the petrous bone used for ancient DNA analysis is 5985–5754 cal BP (5130±25 BP, PSUAMS-9396), a difference too marked to be a marine reservoir effect. The genetic analysis also indicates admixture with people having European farmer- and Levantine-related ancestry, providing independent support for a later date. These results suggest an alternative scenario in which the site continued to be used in post-Capsian times, at least as a place for Neolithic people to bury their dead. This practice is attested at other Capsian sites in the eastern Maghreb [27].

Interestingly, earlier exploration of the site already hinted at possible activity during the Mid Holocene. A 14C dating obtained from a sample of *Cardium* shell yielded a date of 6400–5750 cal BP (5320±150 BP), which aligns with the one obtained from the Burial 2 individual [71].

### Ancient DNA data preparation

Ancient human skeletal samples (either cochlear portions of petrous bone or teeth; see Table S1; cochleae were isolated from the petrous bone by sandblasting, followed by milling) were drilled for powder in dedicated clean rooms in Vienna, Austria, with standard protocols to minimize possible contamination. We extracted DNA in Boston, MA, USA using a robotic procedure with silica beads [72], also in clean room facilities. From the extracts, we built sequencing libraries in the presence of uracil-DNA glycosylase (UDG) to reduce errors induced by DNA degradation, with either a double-stranded (partial UDG) [73] or single-stranded (USER) [74] preparation (Table S1). We used in-solution hybridization to enrich for molecules overlapping a set of ~1.2 million targeted SNPs in the nuclear genome, together with the mitochondrial genome [75]. We added 7- or 8-bp indexing barcodes [76] and sequenced the libraries in pools on either NextSeq 500 or HiSeq X10 sequencing platforms with 76- or 101-bp paired-end reads.

We assigned the raw sequences to individual libraries based on their barcodes and indices, allowing a maximum of one mismatch. We also merged sequences overlapping by at least 15 bases (with at most one mismatch) using a modified version of SeqPrep v. 1.1 (https://github.com/jstjohn/SeqPrep), retaining the allele call from the base with higher quality score. After trimming barcodes and adapters, we aligned the reads to the mitochondrial reference genome RSRS [77] and the human reference genome (version hg19), using the samse command in BWA [78]. We removed duplicate molecules as well as sequences with mapping quality less than 10 (for nuclear DNA) or 30 (for mitochondrial DNA). Finally, we trimmed 2 bases on each end of aligned sequences to further reduce potential damage artifacts.

For the data used in analyses, we called “pseudo-haploid” genotypes by selecting one allele at random from the sequences covering each targeted SNP (with base quality score of at least 20; sites with no data were marked as missing). We determined the genetic sex of each individual based on counts of sequences aligning to the X and Y chromosomes [79]. We determined Y-chromosome haplogroups using a previously published method [40], based on the YFull YTree phylogeny (v. 8.09; https://www.yfull.com/tree/) with SNPs from ISOGG YBrowse (https://ybrowse.org/). Finally, we determined mitochondrial DNA haplogroups using HaploGrep v. 2.1.1 [80], based on all aligned sequences.

### Quality Control

We used several methods to search for possible evidence of contamination in the ancient DNA data (Table S1). First, we computed the rate of apparent cytosine-to-thymine substitutions in the last position of sequenced molecules (before trimming), with authentic ancient DNA expected to show evidence of such substitutions due to deamination damage. Next, we ensured that all individuals had appropriate sex chromosome sequence ratios (proportion of Y chromosome either < 0.03 for females or > 0.35 for males). Finally, we used two approaches based on observed variation at haploid genome sites (where there should in fact be no variation within a single individual): (1) for mitochondrial DNA, we evaluated mismatch rates using contamMix v. 1.0.1051 [81], and (2) for the X chromosome (in males only), we evaluated mismatch rates using ANGSD [82]. For individual I13901 (ABR), both contamMix and ANGSD indicated the presence of substantial contamination, and thus we only used data for our main analyses from molecules with ancient DNA damage. The contamination estimate reported here for the damage-restricted data is based on mismatch rates from contamMix (coverage was too low to apply ANGSD).

From the published western Maghreb data, we excluded one individual from SKH (skh003) from our qpAdm analyses because of evidence of contamination, and one each from KTG (ktg001) and IAM (IAM.3) from all analyses because of low coverage (< 0.02x).

### Radiocarbon dating

We sent samples of the same skeletal elements used for ancient DNA analysis for accelerator mass spectrometry (AMS) radiocarbon dating (using standard methods) at either the Pennsylvania State University Radiocarbon Laboratory [83, 84] or the University of Georgia CAIS Radiocarbon Dating Laboratory [85]. We calibrated the dates using OxCal (v4.4) [86], and the IntCal20 calibration curve [87]. Two dates were discarded due to suspicion of surface contamination on the samples (no ultrafiltration was applied in the UGAMS laboratory). First, a date of 5030±35 BP (uncalibrated) [UGAMS-72041] for I22852 (Hergla) is (modestly) inconsistent with our previous date of 5130±25 BP [PSUAMS-9396] (p = 0.02), although we note that it does not challenge our assignment of this individual to the Neolithic period. Second, a date of 5317–4970 cal BP [4500±55 BP, UGAMS-72040] for I22862 (DEK) is more recent than any known occupation at the site, and the uncertainty (standard error) of the measurement is unusually large; genetic analyses also do not provide any reason to expect such a recent date.

### Statistical analyses

We performed PCA using smartpca v. 18270 [88], with the options “lsqproject” and “shrinkmode” for projecting ancient individuals onto the axes determined from present-day individuals. Allele-sharing statistics (*f*_4_-statistics; Table S3) were computed using ADMIXTOOLS (qpDstat v. 1152) with the “f4mode” option [89]. For statistics testing relative allele-sharing between Djebba and hunter-gatherers from different parts of Europe, we computed f_4_(X, OUB+IAM; Sicily HG, Y), where X is one of the two Djebba individuals and Y is another hunter-gatherer group. To test for signals of Maghrebi ancestry in Sicily, we computed f_4_(Sicily HG, Northern WHG; Maghrebi, Outgroup), where “Maghrebi” is either Djebba or “OUB+IAM” (one individual from each of OUB and IAM with ~100% Maghrebi ancestry), and “Sicily HG” is either (a) six individuals from Epigravettian contexts; (b) three individuals from Mesolithic contexts; or (c) all nine individuals. Finally, to compare possible sources of European farmer ancestry, we used statistics of the form f_4_(Tunisia, OUB+IAM; Spain EN, Italy N), for five different groupings of the ancient Tunisia individuals and three different Neolithic farmer groups from Italy (Sicily EN [8], Sicily MN [8, 37], and Sardinia MN [37, 38]).

The qpAdm software [6] estimates ancestry proportions for an admixed test individual or group of individuals, based on an input set of proxy sources and outgroups (the test population plus sources make up the “left” set, while the outgroups are the “right” set). The underlying model does not assume that the proxy sources are the exact source populations for the ancestry in the test group, but rather that each component of ancestry in the test group forms a clade with its corresponding proxy source, with respect to the outgroups provided. The software returns the inferred proportions of ancestry related to each of the proxy sources, with standard errors, as well as a p-value for overall model fit. Low p-values indicate that the model is violated, typically because one (or more) of the left populations has some un-modeled ancestry related to one or more of the outgroup populations.

Our “left” population list consisted of the test individual(s) plus proxy sources: either two or three from the set of TAF, “OUB+IAM” (see above), Spain Early Neolithic [31, 35, 36], Sicily hunter-gatherers [29], Spain Mesolithic [30, 31, 33], Sardinia Middle Neolithic [37, 38], Israel Chalcolithic [90], and other Neolithic groups from northern Africa. As right outgroups, we used all of the populations from the following set, provided that they were not present in the left list for a given analysis: Spain Early Neolithic, Spain Mesolithic, Sardinia Middle Neolithic, Israel Chalcolithic, Serbia Mesolithic [34], Turkey Neolithic [31, 40], Iran Neolithic [39, 91], Kenya Pastoral Neolithic [92], and Cameroon Stone-to-Metal Age [93]. We used a threshold of p > 0.05 for considering a model to be successful. We chose this set of outgroups with the goal of helping to constrain the tested models of Maghrebi plus western Eurasian-related ancestry, as well as to avoid potential confounding from different methods of data generation by using only UDG-treated target-capture data in the outgroup set. As noted above, the success (p > 0.05) of a given model does not mean that the proxy sources should be considered as exact representatives for the respective ancestry components in the test group, only that the proxy sources are more closely related (to within our statistical power) to the true sources than are the outgroups.

We performed one special set of qpAdm analyses in which we used present-day Fulani, Laka, and Bulala populations as outgroups to search for possible hints of shared ancestry between the ancient Tunisia individuals (DEK and Hergla) and groups from farther south. Our strategy was to compare baseline qpAdm models with our standard outgroup sets to augmented models in which we added any one of the three present-day populations. In order to reduce batch effect artifacts (given the addition of shotgun-sequenced present-day genomes among the outgroups; see previous paragraph), we used Djebba as our proxy source for Maghrebi ancestry (thus making the data types uniform for the left population set), with the full baseline models being (1) Maghrebi plus European farmer ancestry for DEK1, (2) Maghrebi plus Levantine ancestry for DEK2, and (3) all three sources for Hergla.

We estimated dates of admixture using both ALDER [48] and DATES [49]. ALDER requires at least two individuals in the test population, so to obtain dates for single individuals, we formed pairs consisting of the individual of interest plus the Moroccan OUB individual (who, with ~100% Maghrebi ancestry, should not have recent admixture LD). For DATES, we ran the program in both affine and non-affine modes (allowing or not allowing a non-zero asymptote for the decay of admixture linkage disequilibrium with distance). We report results in units of generations in the past. For ALDER, we also report the inferred amplitude of the decay curve. We consider results to be significant at a threshold of p < 0.05, i.e., |Z| > 1.96 (for ALDER, including both the date of admixture and the amplitude).

### ROH and IBD detection

We used the software *hapROH* [50] to infer runs of homozygosity (ROH) from the genome-wide data, with default settings, using the 1000 Genomes haplotype reference panel. As a minimum coverage level, we applied the recommended threshold of > 400,000 SNPs covered from the core ~1.15 million target set, and we inferred ROH with a minimum length of 4 centiMorgans (cM). To convert ROH distributions to estimates of effective population size (in the absence of apparent familial relatedness between parents), we used a maximum likelihood approach [94], based on inferred ROH segments between 4–8 cM. Reported 95% confidence intervals are based on a grid of log-likelihood scores; for DEK2, the upper bound is effectively infinite.

To infer segments shared identical by descent (IBD) between individuals, we used the software *ancIBD* [51], again with default settings and the 1000 Genomes haplotype reference panel. Genotype probabilities were imputed from *GLIMPSE* [95], as previously described [51]. We set a minimum of 8 cM for IBD detection, with the recommended individual-level quality threshold (at least 70% of imputed SNPs on Chromosome 3 imputed with maximum genotype probability>0.99), together with a minimum coverage threshold of 400,000 SNPs. We tested for sharing between the (four) individuals from this study meeting the thresholds and all of the published ancient individuals from the Allen Ancient DNA Resource (AADR, v54.1) [96], augmented with additional published western Maghreb individuals [12].

## Extended Data

**Table 1:** (TO BECOME EXTENDED DATA): Radiocarbon dates and uniparental markers for newly reported ancient individuals. DEK subgroups 1 and 2 are defined from genetic analysis (see main text). For two undated individuals (“N/A”), we use estimates based on genetics and archaeological context in comparison to existing dates. mtDNA, mitochondrial DNA; Haplogr., haplogroup; Seq. Cov., mean sequencing coverage (on autosomal target sites; note coverage for I13901 is after damage-restriction).

IID	Site	C14 Date	mtDNA	Y Haplogr.	Seq. Cov.
I13901	ABR	N/A (>10000 BP)	U6a6	E1b1b1a1	0.05
I20824	Djebba	8178–8026 cal BP	U6a3+185	Female	0.68
I20825	Djebba	7971–7800 cal BP	U6a	E1b1b1a1	0.39
I22580	DEK (1)	7161–6906 cal BP	U6d	Female	6.43
I22862	DEK (1)	N/A (~7050 BP)	U6d	Female	0.55
I22867	DEK (2)	6888–6678 cal BP	L3f1b+16292	E1b1b1a1	2.94
I22866	DEK (2)	6828–6662 cal BP	U5b2b1	Female	3.44
I22577	DEK (2)	6400–6305 cal BP	U6b	E1b1b1a1	2.36
I22852	Hergla	5985–5754 cal BP	R0a2	T1a1a	0.93

**Extended Data Table 1:** Radiocarbon dating of the different levels of the Afalou Bou Rhummel shelter (from [57, 59]), with hypothesized correspondences between layers from the Arambourg and Hachi excavations (see Methods).

Arambourg stratigraphic level	Hachi stratigraphic layer	Lab code	Date (C14 BP)	Date (Cal BP)
I	III	Ly 3227	11450 ± 230	13580–13106
I	IV	Gif 6532	12020 ± 170	14093–13675
		Ly 3228	12400 ± 230	14907–14097
		Alger 0008	13120 ± 370	16532–15223
III	X	Gif 9637	14910 ± 180	18496–17960

**Extended Data Table 2:** Distribution and types of burials at the Afalou Bou Rhummel site, with hypothesized correspondences between layers from the Arambourg and Hachi excavations (see Methods).

Type of burial	Number of individuals	Excavation	Level of appearance	New denomination
Plural burials	49	Arambourg, (1928– 1930)	Level I (Arambourg)	Burial A
Collective burials	8	Hachi (1983–1993)	Level I (Arambourg), layer IV (Hachi)	Burial B
Double burials (H 28, H 16)	2	Arambourg, (1928–1930)	level III (Arambourg)	Burial C
Individual burial (H IX)	1	Hachi (1983–1993)	Layer X (Hachi), level III (Arambourg)	Burial D
Individual burial (H X)	1	Hachi (1983–1993)	Layer X (Hachi), level III (Arambourg)	Burial E

## Supplementary Material

Supplementary online tables

## Figures and Tables

**Figure 1:**
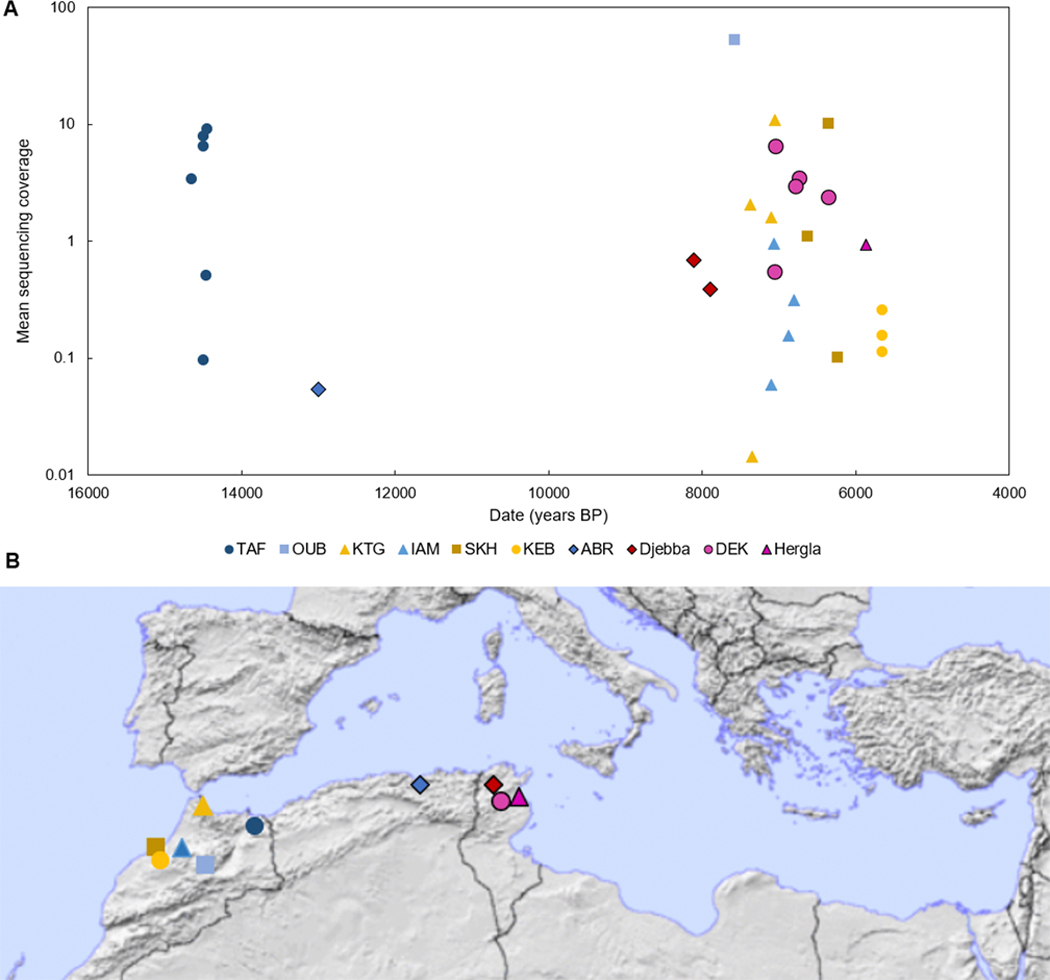
(A) Radiocarbon dates and sequencing coverage (see also Table 1; two undated individuals are estimates only) for previously published and newly reported (black outline) ancient individuals. Note the log scale on the y-axis. Site abbreviations are defined in the main text. (B) Locations of the sites in present-day Tunisia, Algeria, and Morocco. The map is from https://ian.macky.net/pat/.

## Data Availability

The aligned sequences will be made available through the European Nucleotide Archive under accession number ________ [to be made available upon publication]. Genotype data used in analysis will be available at https://reich.hms.harvard.edu/datasets.
